# Conductive Highly Filled Suspensions for an Electrochemical Dispensing Approach to Pattern Full-Area Thin Metal Layers by Physical Vapour Deposition

**DOI:** 10.1038/s41598-020-64105-1

**Published:** 2020-05-04

**Authors:** Katharina Gensowski, Sebastian Tepner, Sebastian Schweigert, Florian Clement, Mathias Kamp, Maximilian Pospischil, Jonas Bartsch

**Affiliations:** Fraunhofer Institute for Solar Energy Systems (ISE), Heidenhofstr. 2, 79110 Freiburg, Germany

**Keywords:** Chemistry, Engineering, Materials science

## Abstract

This paper presents a systematic approach for the development of highly filled suspensions used for an electrochemical dispensing approach. Electrochemical dispensing is an alternative structuring process to locally pattern PVD full-area thin metal layers with the goal to create contacts on solar cells or circuit boards by anodic metal dissolution. Achieving a narrow patterned line width requires a dispensing paste with a high yield stress, a small particle size distribution and a good electrical conductivity. Therefore this work focuses on the formulation and characterization of dispensing pastes in terms of their rheological and electrical properties and their particle size distribution. Furthermore, the printing performance is evaluated in dispensing experiments. In this study, samples with a yield stress above 5000 Pa and an average particle size below 0.4 µm were produced, resulting in dispensed line widths below 100 µm with a high aspect ratio above 0.6. The lack of electrical conductivity was solved by adding KCl solution to the paste, which will add to the ionic conductivity of the NaNO_3_ basis paste formulation. With this approach, printed line widths down to 115 µm and etched line widths below 90 µm at high aspect ratio were achieved on 50 nm aluminum layers.

## Introduction

Today’s metallization of silicon solar cells is dominated by flatbed screen printing because of its robust, fast and cost effective production capabilities. However, the PV industry is not only increasingly demanding for a further optimization of the geometry of printed electrodes, but additionally requests a reduction in production cost of the solar cell metallization step in general^1^. An alternative process to fabricate metal contacts on solar cells as well as on circuit boards is the electrochemical dispensing approach (ECD) in combination with electroplating^2,3^. This approach requires water-based, electrically conductive printing pastes to pattern thin, full-area metal layers by physical vapour deposition (PVD), especially aluminum, copper and silver^4^. The ECD setup consists of a commercially available dispensing machine combined with minor adjustments to provide a necessary electrical circuit. The potential throughput rate of ECD is similar to the state of the art flatbed screen printing and dispensing process of commercial available metal pastes^5,6^. Over the last 15 years the PV industry is demonstrating how a systematic approach for paste development and rheological improvements contributed significantly to a further optimization of printed line geometries. The front-side metallization of silicon solar cells by the flatbed screen printing approach reported printed line widths of up to 150 µm in 2005, improving to reported line widths of only 26 µm by Tepner *et al*. in 2019^6,7^. Furthermore, Pospischil *et al*. reported printed line widths of 65 µm in 2011 and only 17 µm in 2019 for the same silicon solar cell metallization by the parallel dispensing approach^8,9^. These achievements were only possible because the rheological and electrical behavior of industrial silver pastes were improved over the years.

The desired conductive dispensing paste for ECD needs to be based on a (preferentially aqueous) solution of electrochemically active species, typically sodium nitrate (NaNO_3_) or sodium chloride (NaCl), which will govern the anodic metal dissolution and provide a certain ionic conductivity. In addition, an optimized rheological behavior is desired in order to ensure printing of narrow lines width at maximum line heights^10^. The ratio of the two, commonly described as the optical aspect ratio, serves the purpose of locally providing sufficient electrochemically active species to increase the capacity and potentially also the rate of etching. Furthermore, the particle size distribution of the developed dispensing paste needs to be optimized to prevent clogging of micro nozzles. In order to guarantee a sufficient conductivity and minimize unwanted side reactions, these pastes should be based on neutral, aqueous solutions. Currently, pastes for electrochemical dispensing which exhibit these requirements are commercially not available.

Therefore, this work focuses on the development and characterization of a variety of dispensing pastes, particularly on rheological, conductivity and particle size distribution measurements. Finally, the printing and etching performance of all pastes was examined during dispensing experiments. Based on the presented empirical results, the interaction between paste components and paste properties is analyzed in order to derive an efficient procedure for their systematic development.

## Method and Materials

**Figure 1:**
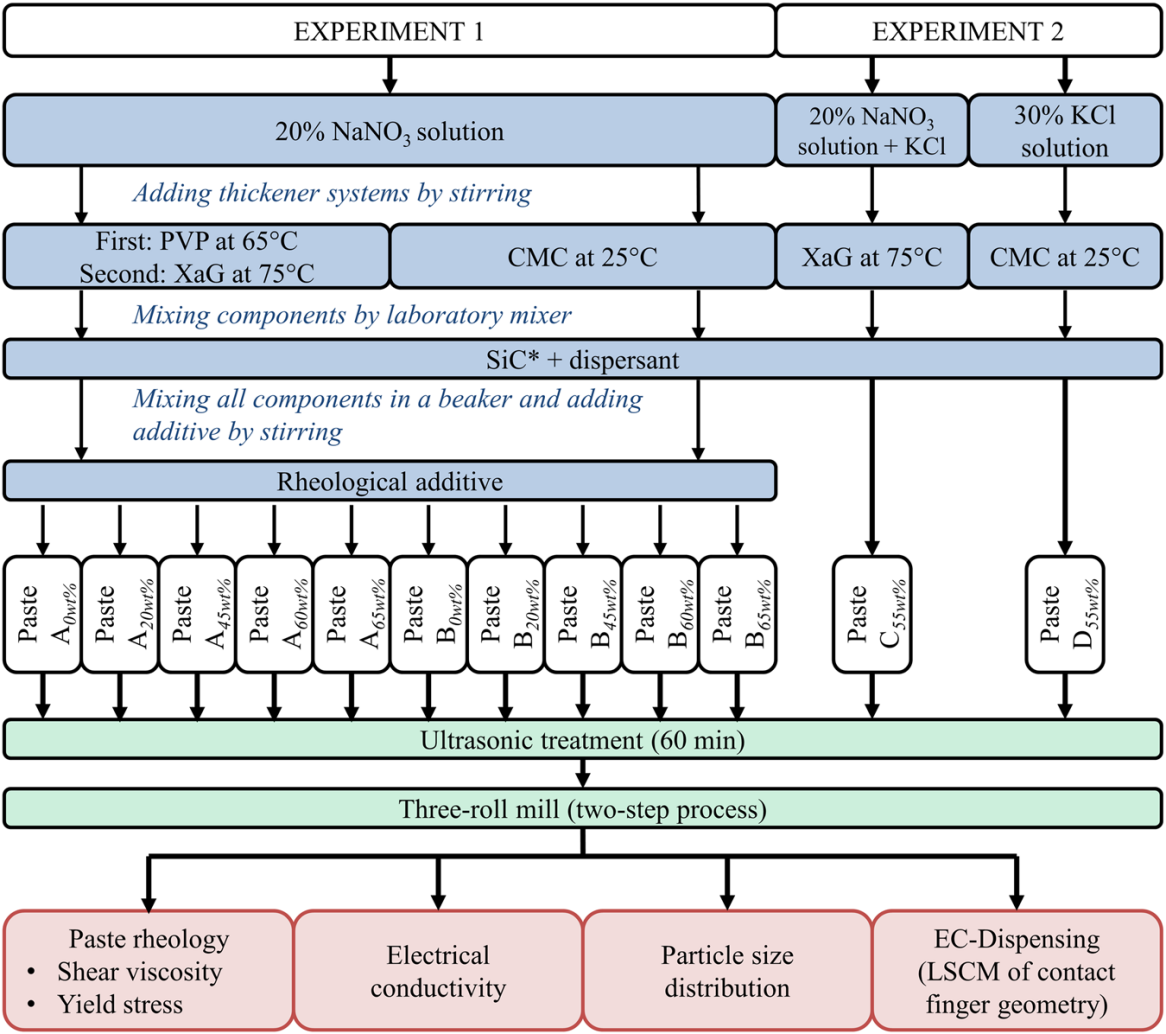
Experimental overview of conductive highly filled suspensions. Schema of the procedure of paste preparation, homogenization and characterization. The different pastes formulations are manufactured by using a stirrer and a laboratory mixer, which may lead to inhomogeneous pastes. As next step, dispensing pastes are homogenized in a two-step process. The suspensions are characterized concerning electrical conductivity, particle size and rheological properties. Finally, ECD experiments are conducted. The paste formulations of experiment 1 and 2 differ concerning the used aqueous base solution, the used thickener system and the silicon carbide (SiC) particle fraction. In total 12 paste formulations (4 groups) are presented in this study (*particles, solid) (CMC – carboxylmethyl cellulose; PVP – polyvinylpyrrolidone; XaG – xanthan gum).

Figure 1 illustrates the procedure of the development of conductive highly filled suspensions and their characterization. The paste formulations are manufactured by using one optimized procedure with a stirrer and a laboratory mixer. The conductive highly filled suspensions (= dispensing pastes) are homogenized in a two-step procedure, ultrasonic treatment and three-roll mill. Subsequently, the pastes are characterized concerning the rheological properties, the particle sizes and the electrical conductivity properties. ECD experiments are conducted with the developed dispensing pastes.

In total, twelve different paste formulations are presented in this paper which differ regarding the used aqueous base solution, the used thickener system and the particle fraction. NaNO_3_ solution, potassium chloride (KCl) solution and a mixture of these two aqueous base solutions are evaluated as electrochemically active solution in the paste formulations. Xanthan gum (XaG), polyvinylpyrrolidone (PVP) or carboxylmethyl cellulose (CMC) thicken the aqueous base solution. The applied silicon carbide (SiC) particles have a mean particle diameter of 0.5 µm. Rheological properties of a suspension depend strongly on the particle fraction^11^. In the first experiment, the silicon carbide (SiC) particle fraction varies between 20 wt% and 65 wt%. For each thickener system, a particle-free dispensing paste acts as reference medium. Based on the results of the first experiment, the paste formulations of the second experiment feature a SiC particle fraction of 55 wt% (Fig. 1).

### Procedure for paste homogenization

After manufacturing, all conductive highly filled suspensions are homogenized in a two-step process which consists of an ultrasonic treatment and a subsequent two-step milling process. The homogenization process increases the uniformity of the paste, because agglomerates are broken up and air inclusions are removed from the printing paste.

The dispensing paste is placed in an ultrasonic bath for 60 minutes. A Sonorex Super from Bandelin (frequency 35 kHz) is used in this work. During this treatment, the number of air bubbles and agglomerated particles are minimized by applying vibrations to a water bath. This causes an ultrasonic field, which transports excess and negative pressure in waves.

Agglomerates and particles are sheared in the roll gap of the three-roll mill (80E, EXAKT Advanced Technology GmbH) in contact mode. A speed of 100 min^−1^ is set. In the first run, the gap between roll 1 and 2 is 25 µm , the gap between roll 2 and 3 15 µm. Subsequently, the paste is milled a second time. For the presented paste homogenization, the chosen gap is 10 µm or 5 µm, respectively.

### EC-Dispensing approach

**Figure 2:**
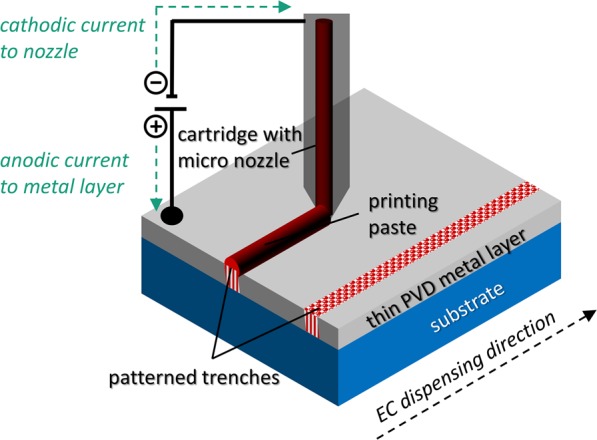
Schematic depiction of electrochemical dispensing during the ongoing patterning of a full-area thin metal layer. The conductive highly filled suspension closes the electric circuit between the nozzle (cathode) and the PVD metal layer (anode) and consequently allows a locally anodic dissolution of the metal.

The dispensing experiment is conducted on a commercially available table robot (Vieweg GmbH), which allows for a precise relative movement between the dispensing nozzle and the substrate in 3 dimensions with an accuracy of σ_x_ = 8 µm in x-direction (parallel towards dispensed lines), σ_y_ = 8 µm in y-direction (orthogonal towards dispensed structures) and σ_z_ = 1 µm in z-direction. The dispensing paste is filled into a cartridge, which is directly connected to the micro nozzle. The volume flow rate of each paste sample is generated by a constant pressure applied to the cartridge. The experiment is done similarly to metallization of silicon solar cells by the parallel dispensing approach, described in literature^5,9,12,13^. Depending on the rheological parameters of each dispensing paste, it is necessary to evaluate the correct pressure in order to ensure a sufficient volume flow rate for a given speed v_x_ of the table robot. The gap in z-direction between the micro nozzle and the substrate is constant at h_disp_ = 100 µm for all experiments. The thickness of the aluminum sheet on the substrate is 50 nm. The diameter of the micro nozzle is varied between 160 µm, 110 µm and 60 µm. Furthermore, an electric potential between the micro nozzle (cathode) and the aluminum sheet (anode) is applied and varied in order to drive a current through the paste thread within the dispensing gap h_disp_ and therefore closing the electrical circuit (Fig. 2).

Nine ECD experiments with a defined parameter variation are performed for each dispensing paste, e.g. three printing speed variations (10 mm/s, 45 mm/s, 70 mm/s), two voltage variations (5 V, 10 V). Line structures with a length of 120 mm are dispensed. Further experiments with the dispensing paste, which shows the most promising printing results, are applied by executing a more detailed parameter variation.

### Characterization methods

#### Measurement of the electrical conductivity

The electrical conductivity of the dispensing pastes is measured by using the TetraCon® 325/S sensor, which is developed specially for emulsions and pastes. Here, four graphite electrodes are arranged in parallel to measure current I and voltage U, from which the electrical conductivity λ is calculated as described in literature^14^. An integrated temperature sensor in the measuring gap quantifies the temperature of the medium. The electrical conductivity is used as the first indication for the etching effect of the suspension, which is one required property for the ECD approach.

#### Measurement of the particle size distribution

The particle size distribution is measured with the LS 13 320 Laser Diffraction Particle Size Analyzer from Beckman Coulter^15,16^. The measuring device works with a scattered light measuring method. The particle sizes are determined according to Fraunhofer theory^17^. For the application of this theory, the particles are assumed to be spherical and must be significantly larger than the wavelength of the monochromatic laser. The distribution density function (volume fraction of the particles with a certain diameter at the total volume) and the particle size distribution (d_x_ values) are the result of this measurement. The d_90_ value indicates that 90% of the particles have this value as maximum particle size or even smaller. The conductive highly filled suspensions have to be diluted with deionized water to realize this measurement. The samples are placed in an ultrasonic bath for 15 min before doing the measurement. The particle size distribution of each sample is determined six times, the measurement time takes 90 s each time. The measurement is supported by using ultrasonic agitation. The particle size (d_90_ value) and the particle distribution are critical for the nozzle diameter to be used and consequently for the patterned structure width and the process stability^13^.

#### Rheological measurements

A commercial rotational rheometer from Anton Paar GmbH, Germany (type MCR302) is used to obtain rheological measurements. For measurements of viscosity and yield stress, a plate-plate geometry is chosen, using an upper plate with a surface roughness R_q_ = 2–4 µm and a diameter d = 25 mm at a measurement gap of h = 500 µm. The shear viscosity for all investigated pastes is measured with a stepwise shear controlled measurement procedure from 0.01 s^−1^ to 1000 s^−1^ with 40 points per decade. Before the beginning of the measurement procedure, the edge of the rotating disk is cleared from exceeded paste. The determination of the apparent yield stress which describes the critical stress at which the paste changes from elastic reversible deformation to irreversible flow, is done similarly with the only difference that a torque (shear stress) controlled measurement procedure from 1 to 10000 Pa with 40 points per decade is chosen. Afterwards, the critical yield stress can be calculated by the tangent intersection method^18^. All measurements are repeated three times at 25 °C and their mean values are presented. Each measurement is done with a new paste sample because extensive pre-shearing has to be avoided.

#### Geometric analysis of printed structures

Measurements of the mean average width w_f_ and the mean average height h_f_ of dispensed structures are done with a commercially available 3D laser scanning confocal microscope LSCM (Lext Series, Olympus) with a magnification of 50–200. Spreading on the substrate is included into all measurements. Each dispensed segment is measured at 5 different randomly chosen positions and the average values are presented.

## Results

### Rheological behavior

**Figure 3:**
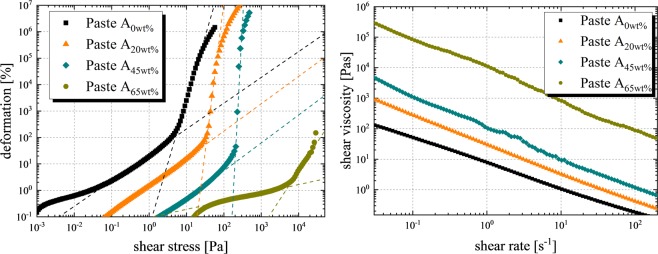
On the left, measurements of the deformation rate over the applied shear stress are shown. Data obtained as described in section *rheological measurements*. The yield stress is determined by the tangent intersection method, well described in literature^18^. The corresponding yielding point increased exponentially with increasing mass share of SiC particles. On the right, measurements of the shear viscosity depending on the mass share of SiC particles for paste A are presented, revealing a similar relationship as the yield stress measurements.

In Fig. 3 (right), the apparent dynamic viscosity for each produced paste of group A is presented, revealing a strong correlation with the mass share of SiC particles. Paste A_0wt%_ has a shear viscosity η of 0.2 Pas ± 0.04 Pas at a shear rate $\mathop{\mathrm{\gamma }}\limits^{.}$ of 100 s^−1^. Increasing the SiC particle proportion to 65 wt% results in a shear viscosity η increase to 87.6 Pas ± 4.6 Pas at a shear rate $\mathop{\mathrm{\gamma }}\limits^{.}$ of 100 s^−1^. For very highly filled suspensions, e.g. 60 wt% or higher, the measurement becomes less stable at high shear rates because of shear banding effects and excessive sample spillage^19^.

The rheological properties of paste C (η = 86.3 Pas ± 5.3 Pas at $\mathop{\mathrm{\gamma }}\limits^{.}$ = 100 s^−1^) and D (η = 3.7 Pas ± 0.7 Pas at $\mathop{\mathrm{\gamma }}\limits^{.}$ = 100 s^−1^) differ significantly, even if the particle proportion of 55 wt% is the same (Table 1). Consequently, the thickener system and the SiC particles show different effects on the rheological behavior of the final paste depending on the used solution. Measurements of the yield stress are shown in Fig. 3 (left), showing a similar exponential correlation with the mass share of contained particles as the viscosity. Paste A_0wt%_ has a yield stress τ_y_ of 5 Pa ± 1 Pa, paste A_65wt%_ has a yield stress τ_y_ of 4100 Pa ± 400 Pa. These characteristics of each paste need to be considered when the performance at the dispensing process is investigated. Dispensing pastes with high viscosity and high yield stress might cause narrow dispensing line widths at high aspect ratio and less paste spreading on the substrate^13^.

### Particle size distribution of dispensing pastes

**Figure 4:**
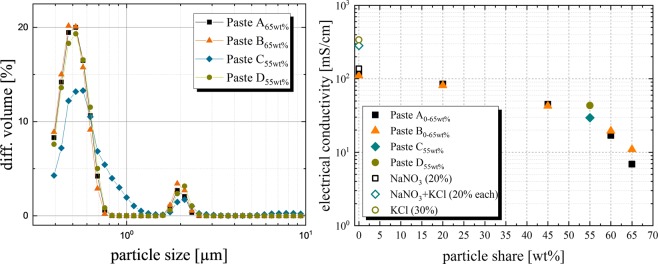
On the left, the volume fraction is plotted over the particle diameter of the corresponding paste formulations. Each curve shows two peaks, where the peak on the left side corresponds to the primary particle size of the used SiC particles. The second peak probably corresponds to agglomerated SiC primary particles. Paste A and B show similar particle distributions, paste C shows the widest particle size distribution. On the right, the electrical conductivity depending on the particle mass share is presented. The various paste components, especially a high proportion of SiC particles, significantly reduce the electrical conductivity of the ionic solution.

Table 1 summarizes the results of the particle measurements of SiC containing paste samples, particle-free formulations were not measured. Paste A_20wt%_ has a d_90_ value of 3.16 µm ± 1.93 µm, paste A_65wt%_ has a d_90_ value of 0.66 µm ± 0.21 µm. The particle distribution of the dispensing paste A_65wt%_ does show two peaks, where the second one is much smaller (Fig. 4 (left, black filled squares)). The second peak might indicate agglomeration of particles. The d_90_ value of the dispensing paste decreases as the SiC content increases (Table 1). The proportion of large particles decreases with increasing SiC particle content, the proportion of small SiC particles increases at the same time. The paste samples of paste B show a similar behavior. The d_90_ values of the dispensing paste C_55wt%_ (4.9 µm ± 0.2 µm) and D_55wt%_ (2.1 µm ± 0.4 µm) are higher compared to the paste formulations of the first experiment.

As mentioned in *rheological behavior*, the different aqueous base solutions result in different paste properties. Paste C_55wt%_ has an increased d_90_ value compared to the other paste samples with similar SiC particle proportion. Therefore, paste C_55wt%_ might show increased tendencies to clog nozzles with small diameters, resulting in a reduced process stability.

### Electrical conductivity of dispensing pastes

The different electrical conductivity values of the dispensing pastes are illustrated in Fig. 4 (right) and are listed in Table 1. The NaNO_3_ solution, which is used for the paste formulations of the first experiment, has an electrical conductivity λ of 137.3 mS/cm. Generally, the different thickener systems with the chosen proportion have a small influence on the electrical conductivity, but the amount of SiC particles can result in an enormous reduction of the electrical conductivity of the dispensing paste. Dispensing paste A_*owt%*_ has an electrical conductivity λ of 114.7 mS/cm whereas dispensing paste A_*65wt%*_ has an electrical conductivity λ of 6.8 mS/cm. This corresponds to a loss of 16.5% (paste A_*owt%*_) or 95.0% (paste A_*65wt%*_), respectively, compared to the NaNO_3_ solution. Similar electrical conductivity values could be determined for the formulations of paste B. The base solutions of paste C (λ = 283.0 mS/cm) and paste D (λ = 338.4 mS/cm) are more electrically conductive compared to the NaNO_3_ solution of pastes A and B (λ = 137.3 mS/cm). The share of 55 wt% SiC particles reduces the electrical conductivity significantly compared to each base solution. The higher electrical conductivity of the used solutions of paste C and D is lost to some extent in the paste formulations. Dispensing paste C_*55wt%*_ has an electrical conductivity λ of 29.5 mS/cm, dispensing paste D_*55wt%*_ of 43.3 mS/cm.

Consequently, the dispensing pastes with low electrical conductivity contain a low proportion of electrochemically active solution and show a reduced etching rate, particularly in combination with fast process speeds. Therefore, the higher conductivity values of paste C and paste D should be advantageous to pattern metal layers by ECD compared to those of paste A. Until now, it is not clear, if a more electrical conductive dispensing paste results in a higher process current and therefore in a higher etching rate. To clarify these uncertainties, a measurement setup has to be installed in the ECD setup in future experiments.

### Dispensing results

**Figure 5:**
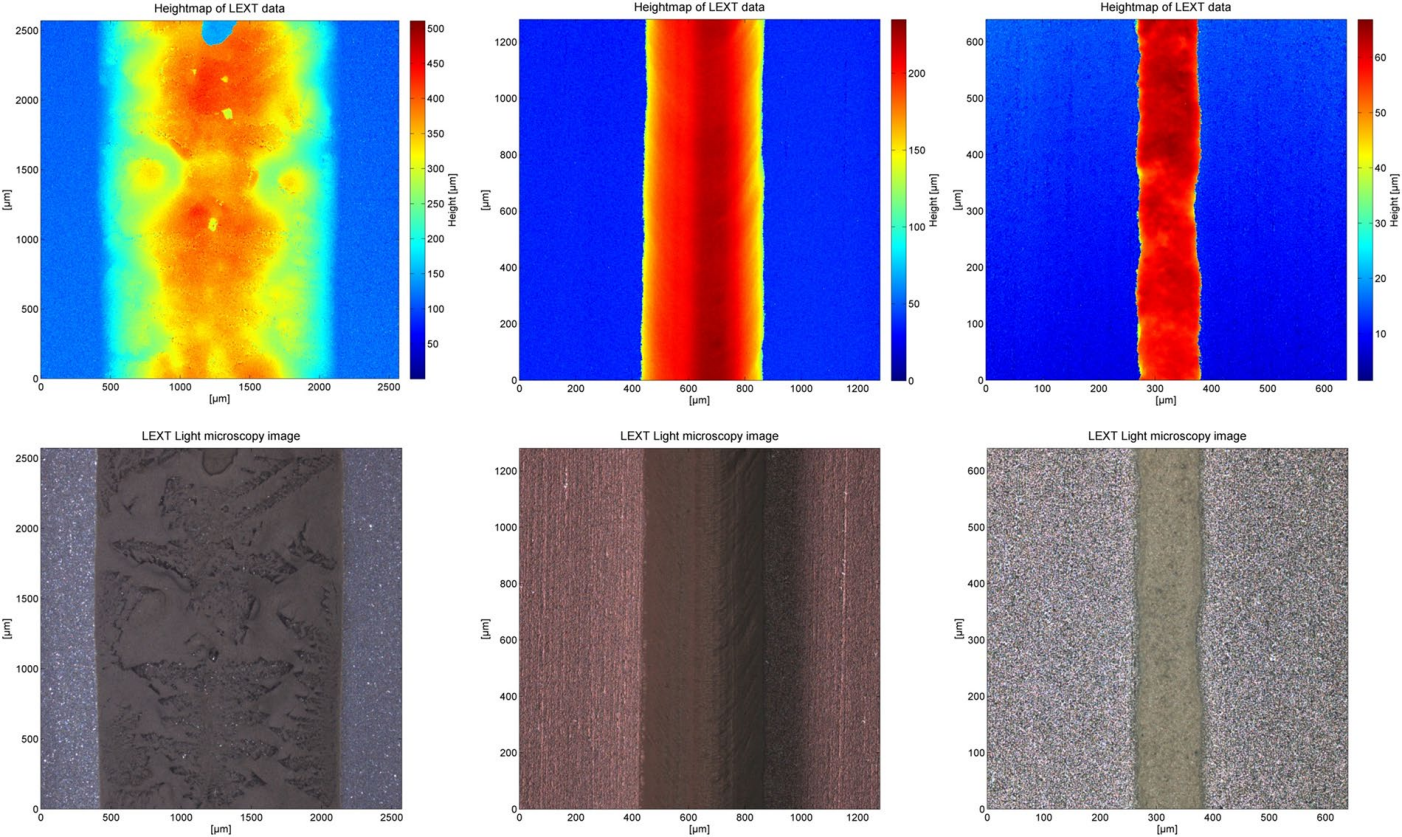
Measurement of the geometry of dispensed lines for different type of investigated paste are presented. All measurements are performed with a 110 µm nozzle. On the z-axis (color) the height at given x-y coordinates is shown. Pictures are taken with a LSCM at a magnification rate of 50 for the left picture, 100 for the center and 200 for the right one. On the left (top) a dispensed line for paste A_20wt%_, in the center (top) for paste A_65wt%_ and on the right (top) for paste C_55wt%_ is presented, revealing how step wise improvements on the paste formulation were achieved. The corresponding etched trenches on 50 nm aluminum layers are presented in the microscope images in the bottom row.

For the investigation of the dispensing performance, the procedure described in section *EC-Dispensing approach* is carried out for all presented paste samples. For each sample, the dispensed line without etching and the etched trench is evaluated by LSCM. Figure 5 presents the geometry of dispensed lines with paste A_20wt%_, paste A_65wt%_ and paste C_55__wt%_ to illustrate how the paste properties have a severe impact on the printing performance. On the left for paste A_20wt%_, the line height is unstable to some extent as drying effects right after the printing step cause an inhomogeneous surface. Furthermore, this sample shows significant spreading due to its low yield stress. The microscope image in the center illustrates a homogeneous dispensed line with a reduced line width and a high aspect ratio because of the higher rheological properties of dispensing paste A_65wt%_. Dispensing paste C_55wt%_ effects again a reduction in line width (Fig. 5 (right)). There, paste C_55wt%_ has an increased yield stress compared to paste A_65wt%_. The line width can also be reduced by optimizing the process parameters, e.g. process speed, distance between nozzle and sample surface.

## Discussion

**Figure 6:**
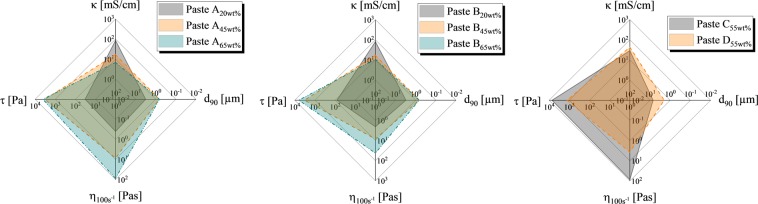
Radar diagrams are used to evaluate the overall performance of a dispensing paste in terms of potential throughput rate, etched line width and capabilities to etch thick aluminum layers. On the left and in the center, the performance of paste A and B for different mass shares of SiC particles are shown, indicating a better printing performance with increased mass share of SiC particles. However, overall electrical conductivity suffers from increased mass share.

In order to gain a deeper understanding of the correlation between rheological and electrical parameters of each paste sample and its respective performance during the electrochemical dispensing process, we present a visualization of the overall performance by radar diagrams in Fig. 6. Each axis represents a critical parameter in log scale. The yield stress (negative direction on the x-axis) gives a strong indication on the dispensed line geometry. Pospischil *et al*. showed that the aspect ratio of printed structures correlates with the yield stress when the surface interaction of the paste and the substrate is kept constant^13^. The shear viscosity at a shear rate of 100 s^−1^ (negative direction on the y-axis) gives a decent indication on the process stability during dispensing (at constant particle size/nozzle diameter ratio) as well as the spreading behavior of each sample. All investigated highly filled suspensions show significant shear thinning behavior as described in section *rheological behavior*. The amount of shear thinning is reduced when the paste is forced through a small dispensing nozzle should stay within a regime where viscous forces still dominate over surface forces, otherwise significant wetting of the nozzle is expected. Furthermore, the shear viscosity at high shear rates and the yield stress both dictate the rate of spreading on the substrate^9,20^. In order to minimize the etched line width, both parameters should be maximized. Additionally, the minimal etched line width is determined by the nozzle diameter. The particle size distribution of a given paste sample is an important indicator for the minimal nozzle diameter which can be used. If the ratio between the nozzle diameter and the particle size become too small, significant clogging is expected. The d_90_ value (90% by volume of all particles feature a size below this value) should therefore be minimized (positive direction on the x-axis). Technically, a small d_90_ value could still lead to clogging of nozzles when significantly bigger particles remain in the paste even at a negligible volume share. Further investigations on the correlation of particle size distributions and the dispensing process stability need to be done in order to prevent clogging of single nozzles completely.

**Figure 7:**
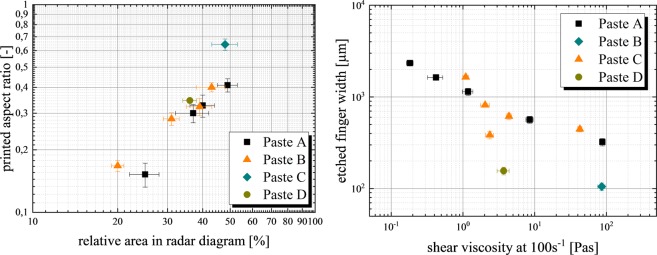
On the left, the relative area covered in the radar diagrams (presented in Fig. 6), is calculated and plotted over the printed aspect ratio. A strong correlation is revealed, indicating that the presented analysis is a suitable method for paste development when high aspect ratios of printed structures are desired. On the right, the correlation of the average etched line width and the shear viscosity at a shear rate of 100 s^−1^ is shown, indicating an exponential relationship. Paste C outperforms the trend significantly due to optimized ratio between electrical conductivity and printing performance.

Finally, the electrical conductivity of each paste sample is plotted in the positive direction of the y-axis because it is one of the most important parameters in order to enable sufficient etching during dispensing. A small electrical conductivity limits the dispensing speed significantly, therefore a paste with maximum conductivity is desired in order to ensure sufficient throughput rates for industrial application. In a previous study, Gensowski *et al*. analyzed different concentrations between 20% and 40% of NaNO_3_ solution with respect to the paste printability. An increased concentrated solution of electrochemically active species leads to an increased electrical conductivity of the paste, but will negatively influence the contour shape of etched lines^10^. In addition, a pretest of this study showed that a KCl solution has a higher electrical conductivity λ (λ_KCl_ = 262 mS/cm at 24 °C) compared to NaCl (λ_NaCl_ = 210 mS/cm at 23 °C) at the same concentration. In Fig. 6 (left) and Fig. 6 (center), the radar diagrams of paste A and B for different volume shares of SiC particles are presented. The different thickener systems of both pastes have little to no effect on the electrical conductivity or the d_90_ value, however the degree of shear thinning behavior differs significantly, indicating a better performance of paste A. In Fig. 6 (right), both paste C and D are presented. The optimized aqueous base solution and thickener result in a promising compromise between etching and printing performance. In order to correlate the combinations of the presented parameters with the outcome of the printing experiments, the resulting area in the radar diagram is calculated and correlated with the aspect ratio of the printed line during dispensing experiments. In Fig. 7 (left), we present this correlation, highlighting that the presented approach to analyze different pastes is suitable for predicting the overall performance in ECD. However, paste C seems to outperform the trend line significantly, resulting in an aspect ratio AR_disp_ of 0.64 at a printed line width w_disp_ of 115 µm and an etched line width w_ECD_ of only 85 µm. The reason behind the importance of the highlighted aspect ratio of the printed structure is the volume of paste material per contact area on the substrate. A maximum volume per contact area is desired in order to enable deep etching of metal layers, when a zincate activation and electroplating on aluminum layers are the follow-up processes^2^. If the aluminum ECD patterned layer thickness is too thin, the zincate activation can remove the entire PVD aluminum layer. This process sequence should be suitable for realizing the metal grid on solar cells. Therefore in future studies, the thickness of these metal layers needs to be varied in order to show that the electrochemical dispensing process provides a significant advantage over the electrochemical screen printing process because it is able to etch deeper into a metal layer at a constant etched line width^21^.

Finally in Fig. 7 (right), we present a direct correlation between the shear viscosity at a shear rate of 100 s^−1^ and the etched line width. Both pastes A and B follow a similar trend, indicating that the use of the same solution and therefore corresponding similar results on electrical conductivity has an overwhelming effect on the expected etched line width. Paste C (τ_y_ = 7210 Pa ± 540 Pa) and D (τ_y_ = 1320 Pa ± 140 Pa) outperform this trend by a factor of 2 to 3, therefore further investigations with these solutions should be carried out. Increasing the volume fraction of particles within the paste will result in an increased shear viscosity and yield stress as described in literature^11,22,23^. In our case paste C has a lower amount of SiC particles compared to paste A, but shows a higher yield stress when using the same primary particles. In addition to the amount of particles, the maximum packing volume fraction, the pH value and the interparticle forces will influence the rheological properties of the highly filled suspensions^24,25^. Mooney^26^ and Krieger and Dougherty^27^ developed first models regarding maximum package fraction and its effect on rheological properties of suspensions. Zhou *et al*. defined the maximum packing fraction as the volume fraction of aggregates in closest-packing at which the relative viscosity approaches infinity^24^. The maximum packing fraction depends mainly on suspended particles shapes and sizes, their particle size distribution, on particle-particle interactions and on hydrodynamic effect. Figure 4 (left) illustrates that paste C has a wider particle size distribution curve compared to the other suspension formulations, meaning that paste C contains more particles of different sizes and agglomerates which consequently will result in an increased packing volume fraction compared to paste A. This is the reason why the d_90_ value of 4.9 µm (paste C) is increased compared to other paste formulations at similar SiC content. It is also possible that the applied thickener system as well as the used solution influence the processing and homogenization of the SiC particles concerning the agglomeration tendency.

In order to increase process stability and throughput rate, future process optimizations needs to take into consideration a parallelization of nozzles by a print head development with an optimized flow geometry e.g. by a CFD simulation approach^13,28^. For paste development, investigations of storage stability, slip behavior within the nozzle as well the parallel print head geometry and suitable particle choices which enables an increase in electrical conductivity by a few orders of magnitude should be considered. All rheological and electrical properties of the highly filled suspensions presented in this paper are summarized in Table 1. This overview also shows the printed and etched line widths of the ECD experiments.

**Table 1:** An overview on the rheological, electrical and dispensing performance of all investigated paste types is presented. Yield stress and viscosity at a shear rate of 100 s^−1^ are determined as described in section rheological measurements. The electrical conductivity is determined as described in section measurements of the electrical conductivity and the d_90_ value for the particle size distribution is determined as described in section measurements of the particle size distribution. Dispensing experiments are done with a dispensing speed of v_disp_ = 45 mm/s. Measurements of printed and etched line width are done with a LSCM as described in section geometric analysis of printe structures.

Paste	Paste properties				ECD results		
	Yield stress τ_y_ [Pa]	viscosity η @100 s^−1^ [Pas]	El. Conductivity λ [mS/cm]	Particle Size d_90_ [µm]	Printed line width w_disp_ [µm]	Printed Aspect Ratio AR_disp_ [-]	Etched line width w_ECD_ [µm]
Paste A_*0wt%*_	5 ± 1	0.2 ± 0.04	114.7	0	2136	0.02	2342
Paste A_*20wt%*_	33 ± 2	0.4 ± 0.05	85.2	3.1 ± 1.9	1752	0.15	1629
Paste A_*45wt%*_	197 ± 23	1.2 ± 0.1	45.0	1.1 ± 0.6	1682	0.33	1139
Paste A_*60wt%*_	3295 ± 240	8.5 ± 0.6	16.9	0.7 ± 0.1	965	0.30	564
Paste A_*65wt%*_	4100 ± 400	87.6 ± 4.6	6.8	0.6 ± 0.1	454	0.41	322
Paste B_*0wt%*_	0	1.1 ± 0.2	110.5	0	1779	0.08	1647
Paste B_*20wt%*_	81 ± 6	2.1 ± 0.3	81.1	3.1 ± 1.4	878	0.17	815
Paste B_*45wt%*_	349 ± 28	2.4 ± 0.3	42.7	0.6 ± 0.1	600	0.28	384
Paste B_*60wt%*_	1096 ± 182	4.4 ± 0.8	19.5	1.0 ± 0.4	797	0.40	614
Paste B_*65wt%*_	6276 ± 574	42.6 ± 2.1	10.9	0.7 ± 0.2	524	0.32	445
Paste C_*55wt%*_	7210 ± 540	86.3 ± 5.3	29.5	4.9 ± 0.2	115	0.64	86
Paste D_*55wt%*_	1320 ± 140	3.7 ± 0.7	43.3	2.1 ± 0.4	440	0.35	156

## Conclusion

In this study we present the development of a conductive highly filled suspension for an electrochemical dispensing process to pattern thin PVD metal layers for silicon solar cell metallization. A variation of twelve different paste samples are manufactured and evaluated in terms of their electrical and rheological behavior as well as their dispensing performance. Furthermore, a method to rate the overall performance of such pastes is presented linking all relevant paste properties with the results of the electrochemical dispensing procedure. It was found, that the use of a 20% NaNO_3_ + KCl solution combined with xanthan gum as a binder at a 55% mass share of SiC particles resulted in the best compromise between printability and etching performance. Printed line structures w_disp_ of 115 µm line width at an aspect ratio AR_disp_ of 0.64 and etched line widths w_ECD_ of only 85 µm were achieved by using this type of dispensing paste.

## References

[1] ITRPV International technology roadmap for photovoltaic. 10th edition. 2018 Results (2019).

[2] Kamp, M. *et al*. Structuring of metal layers by electrochemical screen printing for back-contact solar cells. *IEEE J. Photovoltaics*, 1–7, 10.1109/JPHOTOV.2018.2802201 (2018).

[3] Kamp M (2015). Electrochemical contact separation for pvd aluminum back contact solar cells. Energy Procedia.

[4] Kamp, M., Bartsch, J. & Glatthaar, M. Method for structuring layers of oxidizable materials by means of oxidation and substrate having a structured coating (Aug 18th, 2016).

[5] Pospischil M (2016). High speed dispensing with novel 6” print head. Energy Procedia.

[6] Tepner S (2019). Improving wall slip behavior of silver pastes on screen emulsions for fine line screen printing. Solar Energy Materials and Solar Cells.

[7] Clement, F. *et al*. “Project finale” - screen and screen printing process development for ultra-fine-line contacts below 20µm finger width. 36th European Photovoltaic Solar Energy Conference and Exhibition; 255-258/36th European Photovoltaic Solar Energy Conference and Exhibition; 255–258, 10.4229/EUPVSEC.20192019-2DO.5.1 (2019).

[8] Pospischil, M. *et al*. Applications of parallel dispensing in pv metallization. *AIP Conference Proceedings, 20005*; 10.1063/1.5125870 (2019).

[9] Pospischil M (2011). Investigations of thick-film-paste rheology for dispensing applications. Energy Procedia.

[10] Gensowski, K. *et al*. Paste development for electrochemical screen printing to structure metal layers of back contact solar cells. 33rd European Photovoltaic Solar Energy Conference and Exhibition; 664–669/33rd European Photovoltaic Solar Energy Conference and Exhibition; 664-669, 10.4229/EUPVSEC.20172017-2AV.2.26 (2017).

[11] Zhou Z, Solomon MJ, Scales PJ, Boger DV (1999). The yield stress of concentrated flocculated suspensions of size distributed particles. Journal of Rheology.

[12] Pospischil, M. *et al*. Progress on industrial solar cell front side metallization by parallel dispensing technology. 31st European Photovoltaic Solar Energy Conference and Exhibition; 369-371/31st European Photovoltaic Solar Energy Conference and Exhibition; 369–371, 10.4229/EUPVSEC.20152015-2CO.2.1 (2015).

[13] Pospischil, M. A parallel dispensing system for an improved front surface metallization of silicon solar cells. Fraunhofer Verlag (2017).

[14] Rommel, K. Die kleine leitfähigkeits-fibel: einführung in die konduktometrie für praktiker. *Wiss.-Techn. Werkstätten Weilheim i. OB* (1980).

[15] Beckman Coulter, L. S. 13 320 Laser diffraction particle size analyzer. Instructions for use. Available at, https://www.beckmancoulter.com/wsrportal/techdocs?docname=B05577AB.pdf.

[16] Zimmermann I (1996). Möglichkeiten und grenzen von streulichtmeßverfahren. Chemie Ingenieur Technik.

[17] Nai-Ning W, Hong-Jian Z, Xian-Huang Y (1992). A versatile fraunhofer diffraction and mie scattering based laser particle sizer. Advanced Powder Technology.

[18] Mezger, T. G. The rheology handbook. 4th Edition. For users of rotational and oscillatory rheometers. 4th ed. (Vincentz Network, Hannover, 2014).

[19] Barnes HA (1995). A review of the slip (wall depletion) of polymer solutions, emulsions and particle suspensions in viscometers: its cause, character, and cure. Journal of Non-Newtonian Fluid Mechanics.

[20] Pospischil M (2014). Paste rheology correlating with dispensed finger geometry. IEEE J. Photovoltaics.

[21] Gensowski, K. *et al*. Selective seed layer patterning of pvd metal stacks by electrochemical screen printing for solar cell applications. *Prog Photovolt Res Appl*; 10.1002/pip.3206 (2019).

[22] Wildemuth CR, Williams MC (1984). Viscosity of suspensions modeled with a shear-dependent maximum packing fraction. Rheola Acta.

[23] Ancey C, Jorrot H (2001). Yield stress for particle suspensions within a clay dispersion. Journal of Rheology.

[24] Zhou JZQ, Uhlherr PHT, Luo FT (1995). Yield stress and maximum packing fraction of concentrated suspensions. Rheola Acta.

[25] Yang H-G, Li C-Z, Gu H-C, Fang T-N (2001). Rheological behavior of titanium dioxide suspensions. Journal of Colloid and Interface Science.

[26] Mooney, M. The viscosity of a concentrated suspension of spherical particles, 162–170 (1950).

[27] Krieger IM, Dougherty TJ (1959). A mechanism for non-newtonian flow in suspensions of rigid spheres. Transactions of the Society of Rheology.

[28] Pospischil M (2013). Process development for a high-throughput fine line metallization approach based on dispensing technology. Energy Procedia.

